# Team science criteria and processes for promotion and tenure of Health Science University Faculty

**DOI:** 10.1017/cts.2022.523

**Published:** 2022-12-22

**Authors:** John R. Meurer, Jan Fertig, Orsolya Garrison, Reza Shaker

**Affiliations:** 1 Institute for Health & Equity, Medical College of Wisconsin (MCW), Milwaukee, WI, USA; 2 Humanities, Social Science and Communication Department, Milwaukee School of Engineering, Milwaukee, WI, USA; 3 Clinical and Translational Science Institute of Southeast Wisconsin, Milwaukee, WI, USA

**Keywords:** Team science, academic promotion and tenure, cross-disciplinary research, professional development, team science portfolio

## Abstract

Although team science has expanded with far-reaching benefits, universities generally have not established criteria to recognize its value in faculty promotion and tenure. This paper recommends how institutions might weigh a faculty member’s engagement in team science in the promotion and tenure process. Seventeen team science promotion and tenure criteria are recommended based on four sources – an evaluation framework, effectiveness metrics, collaborative influences, and authorship criteria. Suggestions are made for adaptation of the 17 criteria to committee guidelines, faculty team science portfolios, and the roles of individuals and institutions participating in large, cross-disciplinary research projects. Future research recommendations are advanced.

## Introduction

This paper proposes team science criteria and processes for promotion and tenure of Health Science University faculty. In it, we advance guidelines that can be used by both academic team scientists and the committees evaluating their work. The scope, scale, and complexity of health research have expanded substantially, necessitating the involvement of larger and more academically diverse teams of scientists. Team science is a relatively new and increasingly used approach to solving biomedical questions. During recent decades, multicenter clinical trials, cross-disciplinary investigations, and analyses of big data from research consortia have significantly increased [1]. Since the 1970s, the average number of authors per MEDLINE/PubMed citation has increased from 2 to 6, with a concomitant increase in multi-PI grants [2]. Large team endeavors have been gradually crowding out studies with smaller team composition [3]. Teams increasingly dominate the production of scientific knowledge [4]. There has been an accompanying increase in the size of authoring teams as individual scientists, funders, and universities have sought to investigate multifaceted problems by engaging more individuals [5]. Published work from the science of team science can offer insight into needed changes in health science research. Team science may increase research productivity measured in the number of collaborations, publications, and patents. Multi-investigator centers may lead to an increase in research funding competitiveness. Cross-disciplinary teamwork may foster diverse perspectives and novel approaches.

Team science is a collaborative endeavor that unites expertise from numerous researchers and disciplines to address important research questions from multiple perspectives. A *team scientist* is a collaborative researcher who uses a cross-disciplinary approach to inquiry that leverages the strengths and expertise of professionals trained in different fields. Although collaborative, a team scientist is also a scholarly leader needed to make substantial contributions to solve a joint problem. Team scientists at health science universities engage in collaborative research that applies multi-, inter-, or transdisciplinary tools to the collective solution of complex biomedical and community health problems.

Team science approaches can efficiently and effectively address research questions that involve complex issues. An ensemble that conducts such work may be comprised of clinicians and patients; basic, clinical, translational and community researchers; and health care system representatives, each having unique expertise needed for each task. For example, the National Cancer Institute Transdisciplinary Research on Energetics and Cancer initiative integrates social, behavioral, and biologic sciences to address obesity, physical inactivity, and poor nutrition with the goal of preventing and controlling cancer. The problems to solve and approaches to address them are growing in complexity and therefore require larger and more multifaceted cross-disciplinary teams. These efforts will ultimately generate a deeper understanding of important issues and more efficiently produce scientific discoveries that are readily available to a greater number of communities. For this reason, it is imperative to examine issues of promotion and tenure of faculty researchers who are likely to be at the forefront of such efforts.

Universities need to modify their current systems and the process of appointment, promotion and tenure needs to adapt to keep pace with the ways in which health mechanisms and interventions are discovered through team science [4]. Lack of acknowledgment of team science contributions has concerning implications, not only for the scientist, but for the entire academic community and beyond. Success is less likely if those who engage in team science are viewed, treated, or feel as second-class scholars in the promotion and tenure process [4]. Moreover, a lack of inclusion of team science principles in promotion and tenure documents may discourage scientists from participating in teams that could help them to develop their research skills, increase their productivity, and provide mentorship [6]. The need to be recognized for individual accomplishments, as traditionally defined, may result in faculty choosing to withhold valuable contributions due to equity issues. Universities that de-incentivize team participation are not positioned to solve some critical problems. This state of affairs negatively impacts not only the morale of members and effective functioning of science teams but also ultimately the entire scientific community and its ability to address pressing humanitarian concerns. Thus, new promotion and tenure criteria that recognize the value of team science should be adopted.

The traditional metrics used by universities for consideration of promotion and tenure of health science faculty focus on individual accomplishment using criteria for scholarship, teaching, and service. An analysis of an international sample of 92 universities shows that traditional criteria for promotion and tenure such as peer-reviewed publications, authorship, order of author, journal impact factor, grant funding, and national or international reputation are emphasized more than non-traditional criteria such as team science contributions [7]. As a result, team science is undervalued for the promotion to the rank of associate professor, to non-tenure eligible positions, and to roles prioritizing clinical, education, and administrative responsibilities [4]. Scholarship is generally measured by first or senior authorship on peer-reviewed publications and holding the status of principal investigator on extramural grants. Team scientists, however, are often positioned as middle authors on publications or as co-investigators on grants. Promotion and tenure committees may thus view their contributions as less important than those of first authors and principal investigators. Furthermore, traditional metrics do not recognize that scientific innovation increasingly requires collective creativity. Engagement in team science does not mean that individual work is not being produced or cannot be identified. All too often, however, such work fails to garner the recognition that it merits.

In practice, an individual researcher’s contributions may encompass a mixture of independently led and team science-based scholarly contributions or be comprised entirely of team science-based contributions. Any combination of contributions is acceptable provided that individuals are able to demonstrate their leadership and independence. Most importantly, their status as a team scientist may or may not relate to their authorship position on any given publication. Their academic rank should count according to the substance of their contributions and not the order of their authorship on a publication. A portfolio consisting entirely of team science contributions might be a challenge for promotion and tenure committees which is why guidance for use by both team scientists and evaluation committees is vitally needed.

The need for team science criteria for faculty promotion and tenure is widely generalizable. The topic is of broad concern and a serious challenge for the research community. The issue is important to faculty, division chiefs and department chairs, promotion and tenure committees, peer reviewers, and universities. In addition to making an important contribution to the literature on promotion and tenure, this paper fills an important gap in the team science literature by reviewing and addressing team science considerations in the promotion and tenure process. We provide an in-depth background summary of team science in promotion and tenure, then highlight four seminal works that inform the derivation of a new set of 17 criteria essential in a recommended team science portfolio. The purpose of the proposed criteria is to inspire academic institutional expansion of processes for promotion and tenure that better recognize the value of faculty contributions to team science. Finally, we suggest processes to employ the criteria and note needs for future research.

## Background

Team science is research collaboration conducted by multiple individuals in an interdependent fashion [8]. It is a heterogeneous concept that includes multidisciplinary, interdisciplinary, and transdisciplinary types of collaboration [9]. Multidisciplinarity draws on knowledge from different disciplines but stays within their boundaries. Interdisciplinarity analyzes, synthesizes, and harmonizes links between disciplines into a coordinated and coherent whole. Transdisciplinarity integrates the natural, social and health sciences in a humanities context and transcends their traditional boundaries. A transdisciplinary collaborative effort is required to address a scientific challenge that leverages the expertise of professionals, often trained in different fields, to form a holistic approach [10]. For the purposes of this paper, we acknowledge all three meanings of team science as cross-disciplinary or convergence research.

We explored the topic of team science criteria for promotion and tenure in a literature review of PubMed and searches of Google Scholar and Jane Biosemantics using the keywords of *team science, academic promotion and tenure, transdisciplinary research, professional development,* and *team science portfolio*. PubMed had 11,067 articles on team science and 1,847 on academic promotion. A combination of these terms yielded 69 articles. We included articles most relevant to our purpose published in the last 16 years.

A review of the literature confirmed that traditional criteria for academic appointment and promotion consider scholarship, teaching, and service. Determination of rank is based on a balanced evaluation of a faculty member’s achievements and contributions in these areas. Some universities also recognize achievements in clinical practice, administration, and community engagement. There is heterogeneity in types of faculty appointments, in how tenure may be viewed, and in what tenure means in practice. A traditional path to promotion emphasizes research and teaching excellence. Some university research paths may expect a minimum number of first authored publications, a minimum percentage of publications in high-impact journals, and service as principal investigator of a major grant for promotion to associate professor. Other institution educator paths may expect teaching awards and education scholarship for promotion to associate professor. For example, a clinician educator path emphasizes clinical and teaching excellence. Criteria for promotion may require national or global recognition for influencing a scientific field through publications, presentations, and service on study sections and editorial boards.

Tenure is considered a special faculty status and major achievement at a university that indicates excellence in scholarship. A tenured appointment is an indefinite appointment that can be terminated only for cause or under extraordinary circumstances such as financial exigency and program discontinuation [11]. The principal purpose of tenure is to safeguard academic freedom. Tenure provides the foundation for faculty to pursue research and innovation and to draw evidence-based conclusions free from corporate or political pressure.

In a 2005 survey of US medical schools, many institutions had implemented or were considering recognition of interdisciplinary and team science as well as a broader view of scholarship [12]. Between 2002 and 2005, 15 medical schools (12% of the sample) revised their tenure and promotion guidelines to include an emphasis on team science, and another 24 (19%) were actively considering such a change. Although a few of the surveyed schools provided specific policy language, most lacked specific team science criteria in promotion and tenure documents. Tenure policies included financial guarantee, probationary periods, and part-time tenure.

Several other studies demonstrate the wide variability among academic institutions in approaches to considering team science contributions in promotion and tenure decisions. McHale et al reviewed promotion and tenure documents from 57 NIH Clinical and Translational Science Awarded (CTSA) schools of medicine [4]. Team science was considered to a larger degree for those being promoted to associate professor than to professor, was weighted more heavily for non-tenure-eligible than for tenure-eligible positions, and was given greater consideration for roles prioritizing clinical, education, and administrative responsibilities than those that prioritized research. Guidance for documenting team science accomplishments was more explicit for roles that prioritized research than for those that did not. In a 2011 survey of 58 community engagement core members at 37 of 60 CTSA-funded institutions, Nokes and colleagues demonstrated that team science may include community partners and community-engaged research [13]. About half of those surveyed reported support for community-engaged scholarship and its inclusion in the academic decision processes of rank and tenure, since the CTSA requires a community engagement component. Alperin et al analyzed promotion and tenure documents from 129 universities in the USA and Canada [14]. A large portion of the documents noted public and community service but disregard non-traditional research in partnership with community members. Finally, Brody et al analyzed the promotion and tenure policies of 17 US-based research-intensive nursing schools with over $2 million in NIH funding [6]. They found that only 8 of 17 documents included any reference to team science principles.

Although a significant subset of health science universities has likely made progress in recognizing team science in their rank and tenure processes, their approaches and level of success are not widely disseminated in the published literature or on websites. It is for this reason that we offer a set of team science criteria that can be widely shared among universities in faculty rank appointment, promotion, and tenure consideration.

### Recommended Team Science Criteria for Promotion and Tenure

The criteria we recommend for promotion and tenure are based on four influential publications in the team science field. Mazumdar et al shed light on how contributions to team science can be evaluated [15]. The National Academies of Sciences, Engineering and Medicine (NASEM) provide recommendations for deriving metrics [8]. Stokols et al discuss how to cultivate a receptive environment for implementation of team science promotion and tenure practices [16]. Finally, the *JAMA* Network journals offer authorship criteria that can be adapted for present purposes [1]. As reflected in the titles of these publications, criterion development varies according to the purpose of the work. In our opinion, these publications are too complicated to lend themselves readily to the development of simple, clear metrics by a given institution. We have therefore synthesized metrics from the four sources to create a list of 17 criteria to guide both faculty member and evaluation committees in the assessment of individual faculty member contributions to team science. Instead of providing a deep analysis of these references here, we briefly discuss each one and the process through which the criteria were synthesized. This effort fills a gap in the literature of team science and faculty development, as no such list of metrics currently exists.

In the first of the four publications, Mazumdar et al offer recommendations to promotion committees and department chairs for evaluating team scientists [15]. From their experience, they recommend that: 1) contributions to team-based scholarship, education and service need to be assessed and given substantial weight; 2) evaluations should be founded on well-articulated criteria for assessing the stature and accomplishments of team scientists; 3) mechanisms for collecting evaluative data must be developed and implemented at the institutional level requiring a change in policy and systems; and 4) faculty scientific contributions should be assessed as input into the design of research protocols and grant applications; planning, directing, and conducting data analyses; and input into the development of manuscripts. Although Mazumdar and colleagues provide an essential framework for evaluating team-based research, teaching and service, their charts of major, moderate, and minor activities, sample comments, and additional needed information may be too complex for individual faculty, department chairs, reviewers, and rank and tenure committees to consider. They also do not reference the three other publications noted below which add important perspectives on team science promotion criteria.

Second, in their publication, *Enhancing the Effectiveness of Team Science,* the National Academies of Sciences, Engineering and Medicine (NASEM) recommend good practices and metrics for evaluating team science effectiveness that can be considered in establishing criteria for promotion and tenure [8]. The Academies recommend that leaders of research teams apply specific methods to guide team composition; partner with team-training researchers to create and evaluate professional development opportunities for science teams; and work with universities to create and evaluate science leadership. Additional recommendations include the following: 1) leaders of geographically dispersed science teams and larger groups should provide activities to develop shared knowledge among all participants; 2) universities should collaborate with disciplinary associations to develop principles and criteria for allocating credit for team-based work; 3) Funders should encourage new collaborative models indicating a need for support through the provision of resources; and 4) foundations should require grant applicants for team science-based research to describe collaboration plans and knowledge integration over the life of projects.

In the third publication, Stokols et al present a typology of contextual influences on transdisciplinary collaboration to serve as a basis for deriving promotion and tenure criteria [16]. Their recommendations include as follows: 1) policies and protocols that support successful collaborations; 2) an organizational climate of sharing information, credit, and decision-making responsibilities; 3) strong network linkages between remote sites and available technical support, and provisions for data security and rapid access; 4) interpersonal ability to adapt flexibly to changing task requirements, effective communication among members to develop shared goals, and respect among team members; and 5) a culture that supports collaboration and fosters a willingness devote substantial effort to transdisciplinary activities, provides preparation for the complexities inherent in this collaboration, and encourages participatory, inclusive and empowering leadership styles.

Finally, the *JAMA* Network journals authorship criteria can be adapted to include standards for faculty team science contributions to the intellectual content of grant proposals, research activities, publications, and presentations [1].

Our thesis is that these four seminal sources can be synthesized and distilled to provide a strong foundation for the development of a comprehensive set of team science criteria for promotion and tenure (Table 1). The four references separately highlight the importance of influencing collaboration, measuring team effectiveness, assessing authorship, and evaluating other scientific contributions. Our 17 criteria incorporate suggestions from Mazumdar et al for evaluating team scientists in leadership, service, and academic scholarship including contributions to grants, publications, and research programs. The criteria presented here also include NASEM chapters on team composition and assembly, professional development and education, and leadership in team science. From Stokols et al, our criteria further include social, psychological, and management research on team effectiveness as well as important factors in remote collaborations and community coalitions. Finally, the criteria presented in Table 1 also encompass the *JAMA* list of eight specific substantial contributions to the intellectual content of a paper. Careful, in-depth analysis of the four sources for similarities, differences, and connections enabled the derivation of seventeen strong criteria that will be useful for inclusion in promotion and tenure decisions that affect practitioners of team science.

**Table 1.** Recommended team science criteria for promotion and tenure

Faculty member influences collaboration and team effectiveness
• Co-investigator or consultant substantially influences the direction of grant applications and projects to influence their aims, approach, significance, and innovation to make the proposals fundable and projects impactful
• Mobilize and collaboratively convene the research team and community partners
• Share knowledge or collaborative technologies in dispersed or large teams
• Develop consensus around shared research goals
• Foster respect among team members
• Build network linkages with data security, privacy, and easy access
• Share information, credit, or decision-making responsibilities
• Provide participatory, inclusive, and empowering leadership
• Adapt flexibly to changing tasks
• Contribute to professional and leadership development of research team members
Faculty member contributes to the scientific process and products
• Contribute to the conception of research questions, hypotheses, and specific aims
• Participate in literature review input in the design of research protocols for institutional review board
• Gather data from participants
• Biostatistical study design, data analysis, and interpretation
• Input in manuscript writing and editing and coauthorship of peer-reviewed publications
• Present research findings at local, regional, national, or international meetings
• Support intellectual property ownership, patents, and licensing

The recommended criteria comprise two main categories: Collaboration and Team Effectiveness; and Scientific Contributions. Ten criteria fall into the former category and seven into the latter. The list includes criteria relevant to principal investigator roles such as mobilizing the team, developing goals, fostering respect, sharing credit, providing, and developing leadership, and presenting findings. As another example, several of the criteria are relevant to community-engaged researchers such as mobilizing partners, fostering respect, sharing decision-making, providing inclusion, and developing the collaborative research team.

This hypothetical case illustrates how the team science criteria may be considered in the promotion of an assistant professor active in community-engaged research to associate professor. Some of her portfolio of work is summarized here. In addition to impressive performance in the areas of teaching and service, Dr Scientia has served as co-investigator in grant applications and projects contributing to the conception of research questions and hypotheses. She has performed literature reviews that influenced the direction and impact of grant proposals in a way that clearly made them fundable. She convened community partners to collaborate with the research team and respectfully facilitated the development of shared research goals and pragmatic protocols for survey data collection and privacy protections. In addition to mentoring summer research assistants, Dr Scientia co-chaired regular research meetings with a community partner leader and shared decision-making in adapting the project to changes in the environment. She also served as a reviewing middle coauthor of related manuscripts and led writing endeavors about the community engagement experience. Finally, this team scientist co-presented preliminary findings at local community meetings and presented the results of the projects at national and international scientific conferences, garnering a national reputation for impactful community-engaged research. Through this work, she moved team science and her discipline forward.

### Implementing Team Science Criteria in Promotion and Tenure

Promotion and tenure criteria guidelines should define interdisciplinary research with the goal of recognizing and rewarding team science during promotion and tenure processes [17]. The use of key positions on grants and publications as the primary indicator of research performance, leadership and independence in team science projects should be open, transparent, and standardized [18]. There are a variety of ways in which the recommended team science criteria can be used by stakeholders in the promotion and tenure process. Faculty, department chairs, peer reviewers, and promotion and tenure committees may reflect on this list in evaluating team science contributions. The seventeen criteria can be integrated by university rank and tenure committees into a single master rubric and practical guide. The criteria can also be adapted in the development of questionnaires for team leaders and collaborators to comment on the faculty applicant’s compilation of publications and activities (generally not on a specific publication). Promotion and tenure committees might apply relative weights to the importance of individual criteria that reflect the values of their own institutions for team science productivity. Definitions and descriptions can be added to the criteria. Further, committees might compare these recommendations to their own established team science criteria for promotion and tenure decisions. With input from diverse institutional research leaders, committees might transcend the established practice of giving more weight to first authors and grant principal investigator achievements than to team science contributions in their decisions.

We strongly recommend that each faculty member participating in collaborative research establish a *team science portfolio* to accompany the curriculum vitae to document team science experiences and accomplishments. As an example, an educator portfolio is a valuable accompaniment to a CV that provides faculty with a way to document scholarly teaching and educational scholarship [19]. The team science portfolio highlights collaborative research with a professional goal statement. If a significant proportion of activities are team science contributions, then the faculty member connects their activities to the specific interdisciplinary research promotion and tenure guidelines [17]. The proposed team science criteria can then be used to describe success in influencing collaboration and team effectiveness and contributing to the scientific process and products. The faculty member might note specific contributions in a list of the 17 criteria and provide details about the work not apparent in the CV. The portfolio can thus communicate the importance of contributions to science and highlight the work that could not have been accomplished without their expertise added to the team. For the most impactful publications and substantial grants, statements about roles in the research are useful [20].

Mentors, collaborators, and department chairs can comment on the significance of faculty members team-based contributions to research in their applications for promotion and tenure [20]. In particular, the department chair letter should describe team science contributions based on the criteria. External peer reviews might include research collaborators of the faculty to provide a confidential description of the value of their contributions [17]. Rank and tenure review committees should continually improve methods for evaluation of team science contributions [18]. Application forms and portfolio templates should reflect contributions to team science projects based on the proposed criteria. Finally, faculty should be provided with specific guidelines on how to document team science achievements based on the criteria [4].

A university culture supporting team science requires consistency, alignment, and comprehensiveness at all stages of evaluation, from defining expectations in the initial appointment, to preparing individual candidate CV and portfolio, to incorporating team science criteria in rank and tenure evaluations [21]. University leadership in creating a culture of collaboration and paths for convergence research is essential to academic translation and innovation goals [22]. Institution leaders should directly address barriers to adopting team science criteria in the promotion and tenure process. Academic units must intentionally cultivate team science [23]. Norms should transcend team hierarchy [3]. Incentives within the promotion and tenure system must be aligned with the expressed value of team science [4]. A team science culture should facilitate recruitment and retention of cross-disciplinary researchers.

Resistance to such cultural changes can be expected where there is lack of awareness of the benefits and need for such change, accompanied by insecurity about its potential impact on the academic status quo. Combating such barriers would entail enhancing understanding of how the expansion of the promotion and tenure system is necessary because of the types of problems that science must increasingly address. Furthermore, proponents of such change must educate others about how including team science in the promotion and tenure process benefits the institution in the long run. Researchers who collaborate on larger projects would be more encouraged and therefore experience greater job satisfaction. Adding team science recognition for faculty creates more opportunities and carries great potential for enhancing diversity, inclusion, and equity in academia.

### Needs for Research in Team Science in Promotion and Tenure

While the value of team science is increasingly recognized, research on criteria that can be used to inform rank and tenure decisions is limited. Such research is needed in order to generate stakeholder agreement to advance recognition and reward streams for team science. Further study should target the evaluation of policies and procedures for reviewing investigator promotion based on team science criteria. Focus groups consisting of team scientists, rank and tenure committee members, and other stakeholders are recommended to gain consensus on the recommended criteria. Further research should be initiated in order to promote better understanding of the contextual dynamics of particular fields and types of institutions as well as the effectiveness of changes in rank and tenure processes [21]. Evaluations of the influence of published recommendations on promotion and tenure policies are also needed. Inquiry is into the application of alternative metrics such as H-indices on team science and the impact of collaborative publications on the broader scientific community would greatly enhance understanding of the vital importance of teamwork in contemporary academic scholarship.

This report and the proposed research advance the science of team science by highlighting an important issue that has been invisible relative to other developments in the field, specifically that of lack of recognition of valuable faculty team science contributions to health research. The set of evaluation criteria tailored to university promotion committees are proposed as a starting point toward remediating this deficit and initiating further study.

## Conclusion

Team science plays a vital role in advancing research discovery which reflects impactful scientific progress in many fields and advances in study design and analytic techniques. Team science not only requires rigorous oversight of research methods and reporting but also requires careful attention to promotion and tenure criteria to ensure that the efforts of those who qualify for career advancement are appropriately recognized [1]. Our Clinical and Translational Science Institute of Southeast Wisconsin will promote these team science criteria through our four academic institutional partner rank and tenure committees as well as in faculty leadership groups. We hope other institutes and universities will find these criteria and processes valuable for advancing faculty actively engaged in team science and for evaluating and disseminating their experiences.
